# Percutaneous Ultrasound Guided Implantation of VX2 for Creation of a Rabbit Hepatic Tumor Model

**DOI:** 10.1371/journal.pone.0123888

**Published:** 2015-04-08

**Authors:** Sarah B. White, Jeane Chen, Andrew C. Gordon, Kathleen R. Harris, Jodi R. Nicolai, Derek L. West, Andrew C. Larson

**Affiliations:** 1 Department of Radiology, Division of Interventional Radiology, Medical College of Wisconsin, Milwaukee, Wisconsin, United States of America; 2 Department of Radiology, Northwestern University, Chicago, Illinois, United States of America; 3 Department of Chemical and Biological Engineering, Northwestern University, Evanston, Illinois, United States of America; 4 Department of Biomedical Engineering, Northwestern University, Evanston, Illinois, United States of America; 5 Department of Diagnostic and Interventional Imaging, University of Texas Medical School at Houston, Houston, Texas, United States of America; 6 Robert H. Lurie Comprehensive Cancer Center, Northwestern University, Chicago, Illinois, United States of America; AntiCancer Inc., UNITED STATES

## Abstract

Creation of a VX2 tumor model has traditionally required a laparotomy and surgical implantation of tumor fragments. Open surgical procedures are invasive and require long procedure times and recovery that can result in post-operative morbidity and mortality. The purpose of this study is to report the results of a percutaneous ultrasound guided method for creation of a VX2 model in rabbit livers. A total of 27 New Zealand white rabbits underwent a percutaneous ultrasound guided approach, where a VX2 tumor fragment was implanted in the liver. Magnetic resonance imaging was used to assess for tumor growth and necropsy was performed to determine rates of tract seeding and metastatic disease. Ultrasound guided tumor implantation was successful in all 27 rabbits. One rabbit died 2 days following the implantation procedure. Two rabbits had no tumors seen on follow-up imaging. Therefore, tumor development was seen in 24/26 (92%) rabbits. During the follow-up period, tract seeding was seen in 8% of rabbits and 38% had extra-hepatic metastatic disease. Therefore, percutaneous ultrasound guided tumor implantation safely provides reliable tumor growth for establishing hepatic VX2 tumors in a rabbit model with decreased rates of tract seeding, compared to previously reported methods.

## Introduction

Since its original description in 1933, VX2 has served as a surrogate for tumors involving the liver, esophagus, lung, kidney, uterus and head and neck. [1–8] Because VX2 easily grows when implanted into the liver of rabbits, and it is hypervascular and develops into discrete lesions, this model currently serves as the only large animal model of HCC. This large animal model allows for preclinical research investigating hepatic artery catheterization and site selective delivery of drugs and novel therapeutics to the liver tumors. [9–14]

The current methods of tumor implantation include surgical exposure of the liver, with surgical implantation of tumor fragments, open surgical implantation of VX2 cell suspension and percutaneous injection of minced tumor cells into the liver. [15–20] Open surgical implantation of tumor fragments has proven to be the superior method, with studies reporting success rates ranging from 84–100%, compared to 35–47% success rates when inoculating with cellular suspensions. [14, 15, 21] Because of poor success rates with cell suspension inoculations, other groups developed ultrasound (US) guided methods of implantation with minced tumor cells, and showed 100% technical success rates with decreased associated morbidities with their minimally invasive techniques. [21] Yet another group compared open surgical implantation to US guided percutaneous implantation of tumor fragments, and reported similar success rates in both groups. [22] The objective of this study is to validate the feasibility and efficacy of our modified ultrasound guided approach using implantation of tumor fragments for VX2 liver tumor implantation.

## Materials and Methods

Our study was carried out in strict accordance with the recommendations in the Guide for Care and Use of Laboratory Animal of the National Institutes of Health. The protocol was approved by the Institution Animal Care and Use Committee at Northwestern University (IS 00000363), and all animal care and procedures were performed following institutional guidelines. All New Zealand white rabbits between 6/21/2013 and 5/7/2014 that underwent US guided VX2 tumor implantation were included in this study. The rabbits were females weighing approximately 3.5–4.5 kg.

### Hind Limb Tumor Harvest

A total of 17 New Zealand white rabbits had VX2 samples innoculated into the hind limbs and served as donors for liver tumor implants and for propagation of the VX2 tumor strain. Donor rabbits were anesthetized with an intramuscular injection of ketamine hydrochloride 44mg/kg (Ketaset, Fort Dodge Animal Health, Fort Dodge, IN) and xylazine 3-5mg/kg (AnaSed, Lloyd Laboratories, Shenandoah, IA). Bilateral hind limbs were shaved and disinfected with povidone-iodine 5% (Purdue Products, LP, Stamford, CT). Then, tumors in the hind limbs were explanted and the rabbit was euthanized with sodium pentobarbital 150–200 mg/kg (Euthasol, Virbac Animal Health, Fort Worth, TX). One of the tumors was immediately processed, and the other was placed in normal saline for eventual liver tumor implantation.

In order to process the tumor for propagation, the VX2 tumor specimen was placed in a petri dish and the excess tissue removed. The tumor was rinsed with RPMI 1650 media (Life Technologies, Brand Island, NY) and the necrotic portions discarded. Using a surgical blade, tumor cells were scrapped from the surrounding tissue. The cells were then filtered through a 40 micrometer mesh strainer (BD Biosciences, San Jose, CA) and centrifuged into a pellet. The supernatant was discarded and the cells were re-suspended in a 1:1 ratio with methylcellulose (StemCell Technologies, British Columbia, Canada) and kept on ice.

### Hind Limb Implantation

A naïve rabbit was anesthetized with ketamine and xylazine, as described above. The hind limbs of the naïve rabbit were shaved and disinfected using 70% alcohol. Using an 18 gauge needle, 0.5–1.0 mL of the cellular tumor suspension was injected deep within the gluteal muscles. The tumors were then allowed to grow until palpable (approximately 4–5 cm).

### Ultrasound Guided Implantation of VX2 Tumors into Liver Parenchyma

**Fig 1:**
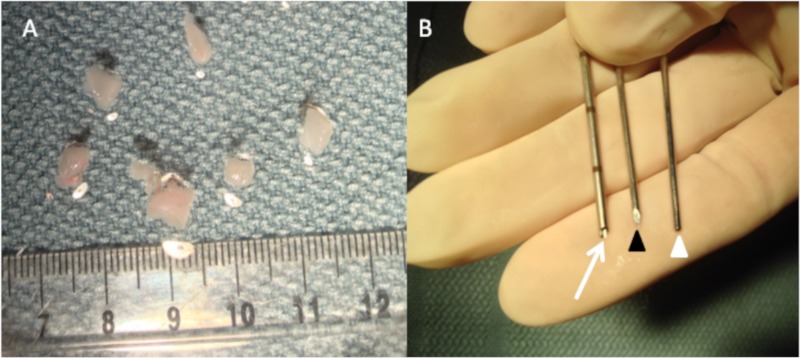
Tumor preparation. (A) Hind limb tumors were placed in a sterile petri dish and sliced into small tumor fragments (3-4mm^3^). (B) A 17 gauge coaxial introducer has a hollow core (white arrow), with two inner stylets, sharp (black arrow head) and blunt (white arrow head).

**Fig 2:**
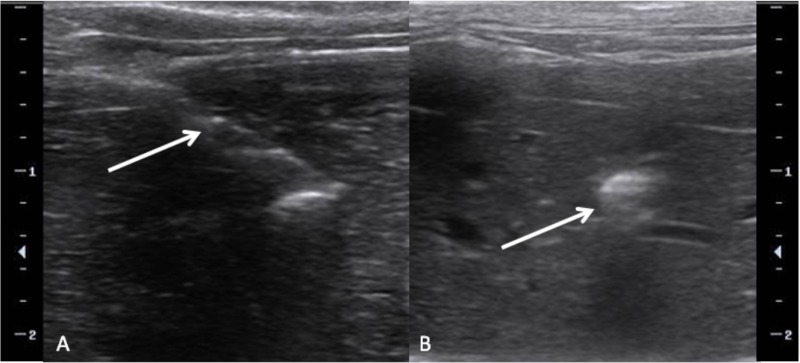
Ultrasound guided implantation. (A) Using ultrasound guidance, a 17 gauge coaxial introducer (arrow) is advanced into the liver. The sharp inner stylet is removed and a small tumor fragment (3-4mm^3^) is placed in the hub of the introducer and pushed with the blunt stylet. (B) Post implantation US image reveals a small focus of hyperechogenicity (arrow) representing the tumor fragment and air.

A total of 27 New Zealand white rabbits had VX2 tumor fragments implanted into the liver parenchyma under ultrasound guidance. Naïve rabbits were anesthetized with an intramuscular injection of ketamine and xylazine, as previously described. Anesthesia was maintained with 2–3% inhaled isofluorane (Piramel Healthcare, Bethlehem, PA), as needed. The abdomen was shaved and a preliminary ultrasound (Mindray M7, Midray Medical Intl Ltd, Shenzhen, China) was performed with a L14-6S transducer for visualization of the gallbladder, bowel, stomach and to determine the target implantation site within the liver. Next, the tumor that was placed in normal saline was sliced into 3–4 mm^3^ fragments (Fig 1A). Under aseptic conditions, a small 1 mm incision was made in the skin using an 11 blade. A 17 gauge 7.8 cm coaxial introducer (C.R. Bard, Inc, Tempe, AZ) was utilized and has a hollow core and a sharp and blunt inner stylet (Fig 1B). Then, the coaxial introducer with the sharp inner stylet was inserted into the liver via a subcostal approach (Fig 2A) under direct ultrasound guidance. The sharp inner stylet was removed and a small tumor fragment (3–4 mm^3^) was pushed through the introducer with the blunt stylet into the liver. Once the tumor fragment was implanted, a focus of hyperechogenicity was seen representing a combination of the implanted tumor fragment and air (Fig 2B). The introducer was then removed and flushed to ensure that the fragment had been implanted into the liver. In cases of residual tumor within the introducer, an additional US-guided puncture was performed. The desired outcome was successful implantation of two tumor fragments into the liver in each rabbit. Post implantation ultrasound was performed to assess for complications including bleeding.

After the procedure, the anesthetic was reversed with yohimbine 0.5 mg/kg (Lloyd Laboratories). Meloxicam 0.2 mg/kg (Norbrook Laboratories Ltd., Newry, Northern Ireland) was administered for pain. Once the rabbits recovered from anesthesia and were in the sternal position, they were returned to their housing. The rabbits were evaluated daily for pain, lethargy, appetite and mobility.

### Follow-up Imaging

**Fig 3:**
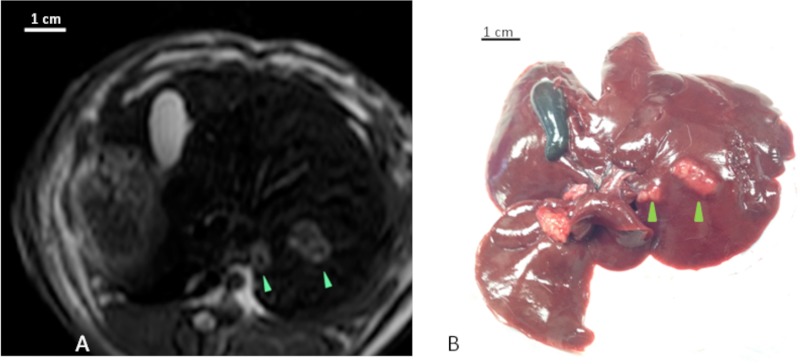
MRI and Gross Pathology. (A) Axial T2 weighted MRI image demonstrates two T2 hyperintense lesions in the left hepatic lobe (arrow heads). (B) Explanted gross specimen reveals tumors corresponding to the lesions seen on MRI (arrow heads).

Magnetic resonance imaging (MRI) (7T Bruker Clinscan, Billerica, MA) was performed 2 weeks post-implantation to assess for tumor growth (Fig 3A) with coronal and axial T_2_-weighted sequences (TR: 2500–3000 ms, TE: 30 ms, Slice Thickness: 1.5 mm, FOV: 130x130 mm, Matrix: 98x128) with an animal respiratory gating system (Model 1025, SA Instruments, Stony Brook, NY). MRIs were performed weekly until tumors reached 1 cm in size. Size measurements were made on follow-up imaging in the anterior-posterior (AP), cranio-caudal (CC), and medial-lateral (ML) orientations.

### Necropsy

**Fig 4:**
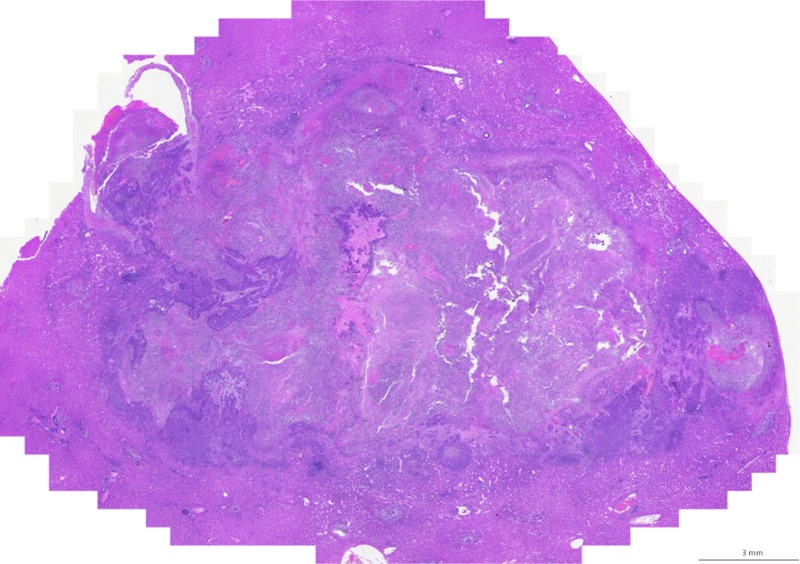
Histology. H&E staining was performed confirming tumors within the liver.

At necropsy, the livers were explanted and assessed for tumor size and location (Fig 3B). The rabbits were also assessed for the presence of metastatic disease on gross pathological examination and size, location and extent of disease were recorded. The liver tumors were then sectioned and sent for hematoxylin and eosin (H&E) staining (Fig 4).

## Results

### Hind Limb Implantation

The hind limb VX2 rabbits required an average of 19.6 ± 5.4 days to reach approximately 4–5 cm in diameter (palpable nodules). No procedural complications or adverse events were observed in any of the 17 rabbits.

### Ultrasound Guided Implantation of VX2 Tumors into Liver Parenchyma

Technical success, or successful implantation of two tumor fragments into the liver in each rabbit, was achieved in all 27 rabbits. These procedures were well tolerated with no immediate complications observed. Imaging follow-up was obtained in 26 (96%) of the 27 rabbits. Imaging follow-up could not be obtained in one rabbit, because it died 2 days after implantation. A necropsy was performed and revealed no definite cause of death. Of the 26 rabbits with imaging follow-up, 5 (19%) had 3 or more tumors, 13 (50%) had 2 tumors and 6 (23%) had one tumor visible on follow-up MRI. In two rabbits (8%), no tumors were ever visualized on follow-up imaging; however, necropsy revealed two sub-centimeter hepatic tumors in one rabbit, and the second rabbit had two tumors in the muscles of the anterior abdominal wall. Therefore, 24/26 (92%) of rabbits implanted harbored tumors visible on MRI imaging. Tract seeding was seen in 2/26 rabbits (8%). Of the 48 tumors that were depicted under MRI, the mean tumor diameter in AP dimension was 1.23 ± 0.55 cm, in the CC dimension 1.0 ± 0.46 cm and in ML dimension 1.31 ± 0.57 cm with the average time of growth of 20.1 ± 8.7 days.

After the rabbits were sacrificed, gross pathology and histology was performed. Liver histology demonstrates a hetergenous appearance to the tumor (Fig 4). Gross pathology indicated that of the 26 rabbits, 10 (38%) had metastases to other sites, primarily to the omentum (n = 2), peritoneal cavity (n = 2), micrometastases to the lungs (n = 6) and intra hepatic metastases (n = 5). Of the 10 rabbits that had metastases, the mean hepatic tumor size was AP 1.33 ± 0.52 cm, CC 1.02 ± 0.39 cm ML 1.36 ± 0.43 cm and the average time of tumor growth was 21.0 ± 12.1 days. Of the 16 rabbits without metastases, the mean hepatic tumor size was AP 1.21 ± 0.54 cm, CC 1.07 ± 0.54 cm ML 1.31 ± 0.66 cm and the average time of tumor growth was 20.2 ± 6.1 days (Table 1).

**Table 1:** Mean tumor growth rates.

	Mean AP diameter (cm)	Mean CC diameter (cm)	Mean ML diameter (cm)	Mean Time of Tumor Growth (days)
Non metastatic disease	1.21±.54	1.07±.55	1.31±.66	20.2±.6.1
Metastatic disease	1.33±.52	1.02±.39	1.36±.43	21.0±12.3
All implanted tumors	1.23±.55	1.00±.46	1.31±.57	20.1±.8.7

## Discussion

Intraparenchymal implantation of VX2 is a widely accepted mechanism for creating a model of liver cancer in rabbits. Prior studies showed that infusion of VX2 into the hepatic artery or portal vein resulted in infiltrative tumors.[23] Assessing tumor response with diffuse hepatic involvement can be very challenging. Therefore, injection of VX2 directly into the liver parenchyma has become the preferred method of tumor implantation. In previous work, tumor inoculation with cell suspension yielded only 35–47% success rates. [14, 15] This method was plagued with morbidity and mortality and leakage of tumor cells was reported to be as high as 50% and mortality rates of 22% were reported.[14, 15] Surgical implantation of tumor fragments resulted in higher rates of success in tumor growth at 84–100%.[14, 15, 21] Though this technique had high success rates, open surgical implantation had been reported to require double the procedural time vs. US guided implantation (15 vs. 7 minutes, p < 0.0001) in one study and 21.5 vs. 16.9 minutes in a second study.[21, 22] US guided implantation was shown to have no resultant infections, while surgical implantation had an infection rates of 8.3%.[21] Additionally, surgical implantation yields rates of extra-hepatic metastases of 50% vs. our reported rate of 38%.[24] Though seemingly superior, the rate of tract seeding in US guided techniques using minced tumor cells was reported to be as high as 20.8% compared to 8.3% in open surgical implantation.[21] Tract seeding is significant in that it results in tumors growing in the abdominal wall, which can be painful to the rabbits and require aggressive pain medications and/or early termination. It can also result in peritoneal studding, with decreases longevity, disrupting studies aimed to evaluate overall survival. Lastly, it can result in no intrahepatic tumors, making the rabbit unusable for studies aimed at treating liver tumors.

In our series, we have modified the previously described US guided technique and implant single 3–4 mm^3^ tumor fragments (total of 2 in each rabbit) instead of minced tumor fragments. Instead of using minced tumor fragments (which are in a semi-solid state), this technique uses a solid tumor fragment. This technique is similar to that described by Luo et. al, in which 25 rabbits underwent US guided implantation via an 18 gauge needle.[22] The tumor fragment was inserted through the needle followed by a small fragment of gelatin foam. Tract seeding was seen in 1/25 rabbits (4%), 9/25 (36%) had metastatic disease, follow-up imaging was only performed on 5/25 (20%) rabbits. In our series, technical success was achieved in 100% of the rabbits and 92% had tumors seen on follow-up MRI imaging. No post-operative infections were seen and tract seeding was only seen in 8%. Gelatin foam was not used in our study due to the initial concern for increased rates of tract seeding and/or infection. Tumor sizes and growth rates were similar in this cohort as with prior studies reported in the literature. In addition, the tumor heterogeneity see in our histology is similar to histology sample seem from other implantation methods. Studies over the last decade have demonstrated that although the micro vessel density in VX2 tumors in heterogenous, the vasculature of the tumor may differ substantially at different stages of growth. At the earlier stages of the tumor's growth, more blood vessels were shown in the peripheral rim of the tumor. This could explain the rapid tumor growth when tumor fragments are implanted. [15]

Implanting tumor fragments has additional benefits. Orthotopic tumor implantation of HCC into a nude mouse model of HCC and other similar tumor fragment implantations have been shown to more genuinely reflect clinical cancer. [25, 26] In addition, investigators have expressed green fluorescent protein (GFP) in animal models of disease in order to visualize tumors.[27] A group in Japan has developed a VX2 cell line that expresses GFP, and was able to propagate the line in rabbit hind limbs. [28] Given that cell infusion can lead to peritoneal seeding, our technique would be useful with this animal model, as less peritoneal seeding is seen, thereby, leaving little peritoneal contamination of the peritoneal cavity with GFP.

There were several limitations in our study. First, this study only includes a small number of rabbits (n = 27). Second, because this technique required fewer resources, it was used exclusively for tumor implantation; therefore, no open surgical group was available for comparison. In conclusion, this study provides additional validation for a percutaneous ultrasound guided method that can safely provide reliable tumor growth for establishing hepatic VX2 tumors in a rabbit model with decreased rates of tract seeding.
